# Oxidative Stress and Inflammatory Biomarkers Are Related to High Intake of Ultra-Processed Food in Old Adults with Metabolic Syndrome

**DOI:** 10.3390/antiox12081532

**Published:** 2023-07-31

**Authors:** Maria Magdalena Quetglas-Llabrés, Margalida Monserrat-Mesquida, Cristina Bouzas, David Mateos, Lucía Ugarriza, Cristina Gómez, Josep A. Tur, Antoni Sureda

**Affiliations:** 1 Research Group on Community Nutrition and Oxidative Stress, University of the Balearic Islands-IUNICS, 07122 Palma de Mallorca, Spain; 2 Health Research Institute of Balearic Islands (IdISBa), 07120 Palma, Spain; 3 Physiopathology of Obesity and Nutrition (CIBEROBN), Instituto de Salud Carlos III, 28029 Madrid, Spain; 4 Clinical Analysis Service, University Hospital Son Espases, 07198 Palma de Mallorca, Spain

**Keywords:** ultra-processed food, NOVA, metabolic syndrome, oxidative stress, inflammation

## Abstract

In the last few decades the consumption of ultra-processed foods (UPFs) worldwide has substantially augmented. Increasing evidence suggests that high UPF consumption is associated with an increase in non-communicable diseases, being overweight, and obesity. The aim of this study was to assess how UPF consumption affects oxidative and inflammatory status in the plasma, neutrophils, and urine of old adults with metabolic syndrome. Participants (n = 92) were classified into two groups according to UPF consumption. Dietary intakes were measured by a validated semi-quantitative 143-item food frequency questionnaire and UPF consumption was determined according to the NOVA classification system. Low UPF consumers showed higher adherence to the Mediterranean diet than high UPF consumers. A high intake of fiber and a high concentration of polyphenols in urine were also observed in subjects with low UPF consumption. Despite the absence of differences in biochemical profile, oxidative and inflammatory biomarkers showed some significant changes. Catalase and superoxide dismutase activities were lower in high UPF consumers, whereas myeloperoxidase activity was higher. ROS production in neutrophils stimulated with zymosan was higher in high UPF consumers than in low UPF consumers. Biomarkers such as xanthine oxidase, tumor necrosis factor α (TNFα), interleukin (IL)-6, IL-15, and leptin levels were higher in participants with high intake of UPF. No differences were found in malondialdehyde and other inflammatory cytokines. The current study evidenced that MetS participants with high UPF consumption have a more pro-oxidant and inflammatory profile than those with low UPF consumption, despite showing similar blood biochemical profiles.

## 1. Introduction

Food consumption patterns have undergone drastic changes worldwide as a result of the globalization process [1]. The generalization of Western diets is partly causing a shift towards unhealthy eating, modifying consumption patterns and lifestyles. The social demand for foods with a longer shelf life and greater palatability has led to the incorporation of natural and artificial ingredients, which affect the nutritional quality of foods [2]. Thus, the consumption of ultra-processed foods (UPFs) has progressively increased, replacing fresh and minimally processed foods in most middle- or high-income countries. Currently, UPF accounts for 50–60% of daily caloric consumption worldwide [3]. 

Thorough analyses have revealed that NOVA is the most precise, consistent, understandable, and practical present classification system. Based on the degree and use of industrial processing, NOVA classifies foods and food products into four categories: unprocessed or minimally processed foods, processed culinary foods, processed foods, and ultra-processed foods. UPFs are defined as foods that have undergone significant industrial processing and are mainly composed of substances used in industrial applications [4]. Examples of UPFs include ready-to-eat foods such as packaged snacks, carbonated soft drinks, candies, ice creams, mass-produced packaged breads and buns, margarines and other spreads, biscuits, pastries, cakes, and breakfast cereals [5]. This kind of food often has a poor nutritional profile, is rich in calories, deficient in fiber and minerals, and has plenty of saturated fats, salt, and sugars, inducing a high glycaemic load [6]. They contain few or no natural foods, such as additives, to extend their useful life, making them highly palatable and profitable [7]. Moreover, compared to minimally processed foods and culinary preparations based on them, UPFs are often more hypercaloric and less satiating [8].

Excessive UPF dietary intakes have been linked to many negative health effects, including non-communicable chronic diseases. The high-energy densities and the significant presence of unhealthy elements, along with low amounts of dietary fiber, contribute to an increased risk of diet-related non-communicable diseases [9]. There is evidence that an increase of 10% in intake of UPFs is associated with an increase of a 15% risk of all-cause death and a 13% risk for type 2 diabetes (T2D) incidence and cardiovascular disease-related mortality [10,11]. A high UPF intake has been linked to dyslipidemia in elderly persons. These atherogenic lipid dysfunctions may be a mediator of the current rise in CVD risk linked to UPF intake [12]. Moreover, it has been observed that older persons with metabolic syndrome (MetS) and who are overweight/obese with high UPF intake showed higher levels of NAFLD-related biomarkers [13]. Moreover, it has been shown that an ultra-processed diet, which is typically high in simple sugars and saturated fats, is associated with the development of alterations in the redox status, altered intestinal microbiota, and high inflammatory response [14]. Consuming high amounts of UPF results in low fiber intake, as the protective fiber layer from grains is removed during ultra-processing. This low-fiber diet has been linked to changes in the composition of gut microbiota, diversity, and epigenetics, causing intestinal dysbiosis [15]. This dysregulation affects the production of short-chain fatty acids and the integrity of the intestinal mucosa, resulting in pro-oxidative and inflammatory processes in the body [14]. 

Keeping in mind the aforementioned factors and the great epidemic of non-communicable diseases suffered by today’s population, the aim of this study was to assess how UPF consumption affects oxidative and inflammatory status in the plasma, neutrophils, and urine of old adults with metabolic syndrome.

## 2. Methods

### 2.1. Study Design and Participants

A total of 92 participants (58.7% men) between 55–75 years old were recruited in Mallorca (Spain). To participate in the study, participants were required to meet three or more of the MetS criteria: triglycerides levels ≥ 150 mg/dL, blood pressure ≥ 130/85 mmHg, fasting serum glucose levels ≥ 100 mg/dL, in men HDL-cholesterol levels < 40 mg/dL and/or waist circumference ≥ 90 cm, and in women HDL-cholesterol levels < 50 mg/dL and/or waist circumference ≥ 80 cm according to the updated harmonized definition of the International Diabetes Federation, the National Heart, Lung, and Blood Institute, and the American Heart Association [16].

Participants were distributed into two groups according to the consumption of ultra-processed foods (UPF) for every 1000 g of consumed food. Firstly, dietary intakes were measured by a validated semi-quantitative 143-item food frequency questionnaire (FFQ) [17]. Each item’s typical serving size was reported, and consumption frequency was noted in nine categories ranging from “never or hardly ever” to “6 times a day”. Then, food items (g/day) were classified by NOVA into four different groups: unprocessed or minimally processed foods, processed culinary ingredients, processed foods, and UPFs [18]. Participants were classified into two groups using the median value of the intake in grams of UPFs (105.9 g UPF per 1000 g total intake per day) as the cut-off. Participants who consumed fewer UPFs than the median were categorized as having low consumption, while those who consumed more UPFs were grouped under high consumption.

The experimental procedure was designed following the Declaration of Helsinki and was revised and approved by the Ethics Committee of Research of the University of the Balearic Islands (CEIC- IB2251/14PI). All participants were informed of the purpose and implications of the study, and informed consent was obtained from all subjects.

### 2.2. Anthropometrics, Dietary Intake, and Physical Activity

Anthropometric measurements were carried out by experienced dietitians. Body weight and the basal metabolism rate were determined and estimated using a Segmental Body Composition Analyzer (Tanita BC-418, Tanita, Tokyo, Japan), respectively. To determine body weight, the participants took off their shoes and dressed in light clothing, and 0.6 kg was subtracted from their weight. Height was measured by keeping the patient’s head in the Frankfort Horizontal Plane position with a mobile anthropometer (Seca 214, SECA Deutschland, Hamburg, Germany). BMI (kg/m^2^) was calculated using the previous measures of weight and height. To size abdominal obesity, an anthropometric tape halfway between the iliac crest and the last rib was used. The waist-to-height ratio (WHtR) was determined as the waist circumference (cm)/height (cm). With a validated semi-automatic oscillometer (Omron HEM, 750CP, Hoofddrop, the Netherlands), blood pressure was measured whilst the patient was sitting.

Dietary intake was measured using a validated semi-quantitative 143-item FFQ [17]. This measurement determines the adherence of the participants to the Mediterranean diet (MedDiet) by a score of 17 items [19] and caloric intake. Using computer software and data from Spanish food composition tables [20,21], energy and nutrient intakes were computed as frequency multiplied by the nutritional composition of a specific portion size for each food item. The dietary inflammatory index (DII) was assessed using the verified FFQ, as previously reported [22]. This metric integrates the impact of 45 items on six inflammatory biomarkers, interleukin-1 (IL-1), IL-4, IL-6, IL-10, tumor necrosis factor α (TNFα), and highly sensitive C-reactive protein (CRP), to assess the inflammatory potential of a diet. Thus, a diet that is more pro-inflammatory is indicated by a positive DII score, while an anti-inflammatory diet is indicated by a negative DII score.

Using metabolic equivalents (METs), the amount of physical activity was calculated while taking into account the rate of energy loss as currently understood [23]. All the subjects discussed the volume of work carried out each week in terms of minutes.

### 2.3. Blood Collection and Analysis

Blood was drawn from the antecubital vein after a 12-h overnight fast in appropriate vacutainers containing ethylenediaminetetraacetic acid (EDTA) as an anticoagulant to acquire plasma and other vacutainers without any anticoagulant to obtain serum. General blood biochemical assays on fasting serum were performed in the clinical laboratory of Son Espases University Hospital (Palma, Spain) using standard enzymatic methods. Glucose, HbA1c, triglycerides, high-density lipoprotein cholesterol (HDL-c), low-density lipoprotein cholesterol (LDL-c), total cholesterol, and uric acid were evaluated in serum using established clinical techniques (Technicon DAX System, Technicon Instruments Corp., Tarrytoen, NY, USA). 

Plasma samples were obtained by centrifuging fresh blood at 1700× *g* at 4 °C for 15 min. Ficoll-Paque PLUS reagent (GE Healthcare Bio-Sciences AB, Uppsala, Sweden) was used to purify neutrophils from fresh whole blood Ficoll was added to tubes containing blood samples in a 1.5:1 ratio, and the tubes were centrifuged at 900× *g* at 4 °C for 30 min. The upper phase, which comprised plasma and Ficoll, was then discarded, while the middle layer of peripheral blood mononuclear cells (PBMCs) and the precipitate containing erythrocytes and neutrophils were recovered. The precipitate, which contained erythrocytes and neutrophils, was incubated in cold water with 0.15 mol/L NH_4_Cl to haemolyze the erythrocytes. Then, the tubes underwent a 750× *g* centrifugation at 4 °C for 10 min, with the supernatant being discarded. The procedure with NH_4_Cl was repeated and, finally, the neutrophil phase at the bottom was washed with phosphate-buffered saline (PBS) at a pH of 7.4.

### 2.4. Collection of Urine Samples 

The participants collected the first morning’s urine themselves in sterile, dry containers. All results for the urine biochemical parameters were normalized using the levels of creatinine measured using Abbott ARCHITECT c16000 (Abbott Diagnostics, Lake Bluff, IL, USA) equipment at the clinical laboratory of Son Espases University Hospital.

### 2.5. Enzymatic Determinations

The plasma activities of the antioxidant enzymes—superoxide dismutase (SOD) and catalase (CAT)—and the prooxidant myeloperoxidase (MPO) were monitored in a Shimadzu UV-2100 spectrophotometer (Shimadzu Corporation, Kyoto, Japan) at 37 °C. CAT activity was assessed following Aebi’s spectrophotometric technique, which relies on the breakdown of H_2_O_2_ at 240 nm [24]. A modification of McCord and Fridovich’s procedure was used to detect SOD activity at 550 nm [25]. MPO activity was lastly ascertained using guaiacol as a substrate by monitoring the development of polymerization products of oxidized guaiacol at 470 nm [26].

### 2.6. Malondialdehyde Assay

Malondialdehyde (MDA) was measured in plasma and urine using a specific colorimetric assay kit (Sigma-Aldrich Marck^®^, St. Louis, MO, USA), and the absorbance was measured at 586 nm following the manufacturer’s instructions. The method is based on the reaction of MDA with n-methyl-2-phenylindole, generating a stable chromophore. Plasma, urine, and standards were reacted with n-methyl-2-phenylindole in acetonitrile:methanol (3:1) and HCl (12 N) at 45 °C for 60 min. A standard curve of known MDA concentrations was used to calculate the concentration in the plasma and urine samples.

### 2.7. Polyphenol Determination

Plasma and urine samples were deproteinized with cold acetone (1:1.2) to determine the content of total phenolics using the method by Folin-Ciocalteau [27] and a standard curve of L-tyrosine for quantification. 

### 2.8. Immunoassay Kits

All immunoassay kits were carried out in plasma samples following the manufacturer’s instructions for use. TNFα and xanthine oxidase (XO) levels were determined using an ELISA kit (Diaclone, Besancon CEDEX, France) and another ELISA kit (Cusabio^®^ Technology LLC, Houston, TX, USA), respectively. Interleukin-1β (IL-1β) and monocyte chemoattractant protein-1 (MCP1) levels were measured using specific ELISA kits (RayBiotech^®^, Parkway Lane, Suite, Norcross, GA, USA). Finally, ghrelin, leptin, resistin, interleukin-6 (IL-6), interleukin-15 (IL-15), and interferon-γ (INF-γ) levels were determined using Human Custom ProcartaPlex^TM^ (Invitrogen by Thermo Fisher Scientific, Bender MedSystems GmbH, Viena, Austria).

### 2.9. Determination of 8-Oxo-7,8-dihydro-guanosine and 8-Oxo-7,8-dihydroguanosine 

The ultra-performance liquid chromatography coupled with tandem mass spectrometry (UPLC-MS/MS; Waters, Milford, MA, USA) approach was used to assess the urinary 8-oxo-7,8-dihydro-guanosine (8-oxodG) and 8-oxo-7,8-dihydroguanosine (8-oxoGuo) concentrations, as previously described [28].

### 2.10. Neutrophils Reactive Oxygen Species Production

Reactive oxygen species (ROS) production by neutrophils was measured after activation with zymosan A (Zym) from *Saccharomyces cerevisiae* (Sigma-Aldrich, St. Louis, MO, USA) and with lipopolysaccharide (LPS) from *Escherichia coli* (Sigma-Aldrich, St. Louis, MO, USA). An amount of 50 μL of fresh neutrophils in suspension (6 × 10^5^ cells) was introduced in a 96-well microplate containing 50 μL of Zym or LPS prepared in 2 mM in PBS, pH 7.4. Then, an indicator, was added into all wells: cell-permeant probe 2,7-dichlorofluorescein-diacetate (DCFH-DA, 61.6 μM in Hanks’ Balanced Salts Medium). The fluorescence (Ex, 480 nm; Em, 530 nm) was registered in an FLx800 Microplate Fluorescence Reader (Biotek Instruments, Inc., Winuschi, VT, USA) at 37 °C for 1 h.

### 2.11. Statistical Analysis

Statistical analyses were carried out using the Statistical Package for Social Sciences (SPSS v.28, IBM Software Group, Chicago, IL, USA). The normal distribution of the data was previously evaluated using the Kolmogorov-Smirnov test. All variables had a normal distribution. A Student’s t-test for unpaired data was performed. All analyses were adjusted by sex. On the other hand, in the case of dichotomous variables, the Pearson χ² test was performed. Results are expressed as the mean ± standard deviation (SD), and *p* < 0.05 was considered statistically significant.

## 3. Results

**Figure 1:**
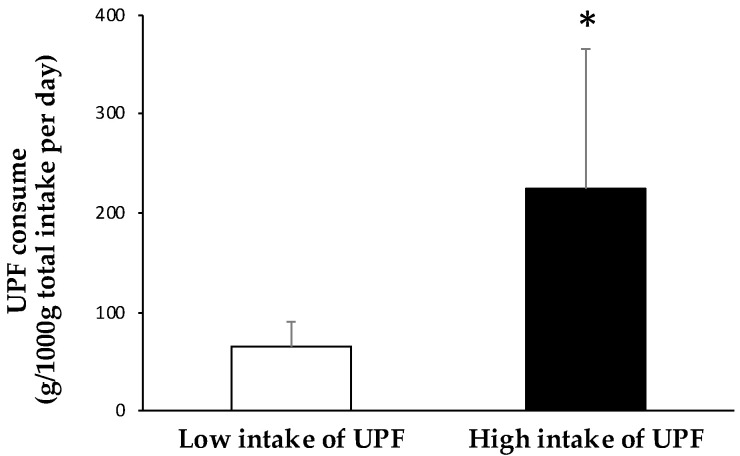
UPFs consumed by the participants of each group represented by grams of UPF intake/1000 g of the total intake per day. Results are presented as mean ± standard deviation (SD). Data points in bold (*) are significant (*p* < 0.05) by Student *t*-test adjusted by sex.

Figure 1 shows the values of UPF intake of subjects according to their low and high consumption. The group with lower UPF intake showed values of 64.5 ± 25.1 g and the group with higher UPF intake showed values of 225 ± 141 g per 1000 g total intake/day. Specifically, the group with a low intake of UPFs consumed 6.5% of this type of food in their daily diet and while the other group consumed 22.5%.

Table 1 displays sample characteristics as well as blood indicators and other lifestyle aspects according to UPF consumption. Patients with a high intake of UPFs showed lower adherence to the Mediterranean diet and higher values of DII than participants with a low intake of UPFs. In both groups, a similar percentage of individuals took antidiabetic, antihypertensive, and lipid-lowering medication. No other differences were found.

Essential nutrients that the body requires to operate and other variables related to UPF intake are shown in Table 2. Participants consuming more UPFs ingest more saturated fatty acids (SFA) and trans-fatty acids (trans-FA) while consuming less fiber per day. No other differences were found across the groups.

Table 3 shows the results from oxidative stress and inflammatory biomarkers in plasma related to UPF consumption. Patients with high UPF consumption showed lower activity of the antioxidant enzymes CAT and SOD than individuals with a low UPF intake. Participants who consumed UPFs showed an increase in MPO activity and XO levels. Regarding inflammation markers, only differences in IL-6 and IL-15 were observed. These two interleukins were more prevalent in individuals who consumed more UPFs. TNFα and leptin levels were higher in participants with higher UPF consumption. Significant variations in polyphenol urine concentration were observed in patients with lower UPF consumption.

**Figure 2:**
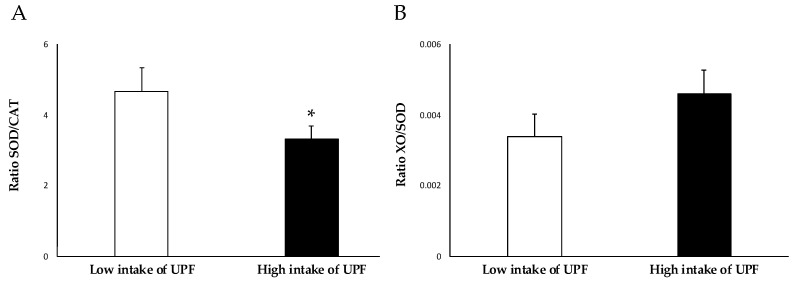
Ratio of (**A**) SOD/CAT (pkat/k) and (**B**) XO/SOD (μg/pkat) according to UPF intake. Results are presented as mean ± standard deviation (SD). Data points in bold (*) are significant (*p* < 0.05) by Student *t*-test adjusted by sex.

**Figure 3:**
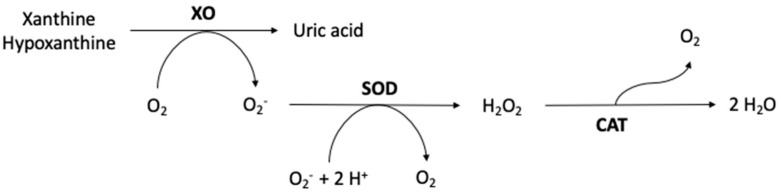
Enzymatic antioxidant defence system.

The relationship between the activity of the enzymes determined above (SOD activity/CAT activity, and XO levels/SOD activity) is depicted in Figure 2 as markers of the redox equilibrium. The study participants with high UPF consumption had a lower SOD/CAT ratio. Although a trend could be observed, no significant changes related to the XO/SOD ratio were found. Figure 3 shows the enzymatic antioxidant defence system. 

**Figure 4:**
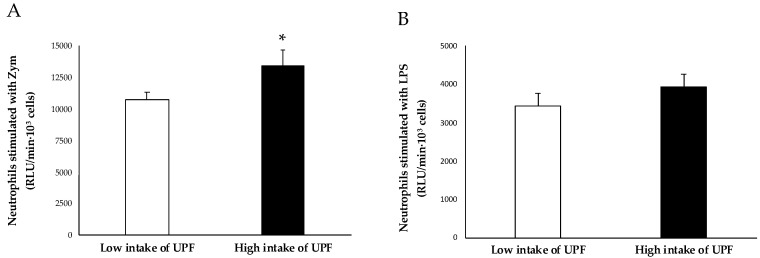
Neutrophils stimulated with (**A**) zymosan A (Zym) or (**B**) lipopolysaccharide (LPS) according to the intake of UPF. Results are presented as mean ± standard deviation (SD). Data points in bold (*) are significant (*p* < 0.05) by Student *t*-test adjusted by sex.

Figure 4 displays the outcomes of neutrophil activation by Zym and LPS that promote ROS production categorized based on the consumption of UPF. Differences can be seen in the production of ROS by Zym-stimulated neutrophils. These significant variations were not seen when ROS production by neutrophils was stimulated by LPS.

## 4. Discussion 

The most outstanding result of this study is that a high UPF intake promotes a more pro-oxidant and pro-inflammatory status in patients with MetS without differences in their anthropometric parameters and biochemical profile. The absence of differences between the two groups in these parameters is mainly because they are two homogeneous groups, characterized by the presence of MetS, as it was the main inclusion criterion. Even so, the intake of a greater amount of UPFs is associated with a greater pro-oxidative and inflammatory state, which may be of clinical interest due to its relationship with cardiovascular risk. In addition, the fact that the factors that determine MetS are within the reference values or close to them could derive from the fact that, regardless of the group in which they were classified, many took antidiabetic, antihypertensive, and lipid-lowering medication [29,30]. The grouping variable was the consumption of UPFs: over/under 105.6 g of UPF consumption per 1000 g intake (approximately 10% of food as UPFs). In this sense, it reported a link between consuming more than 10% of ultra-processed foods and an increased risk of developing cardiovascular diseases, strokes, risk of cancer, and even higher mortality rates [31,32].

The obtained results revealed that participants with high UPF intake showed lower adherence to the Mediterranean diet than participants with lower UPF intake, which was indicative of lower diet quality [33]. Moreover, participants consuming more UPFs displayed a higher DII than those consuming fewer UPFs. These results agree with da Silva A. et al. [34], who reported a direct association between the consumption of processed and UPFs with a high DII, and an inverse association with the consumption of unprocessed or minimally processed foods. Despite observing a trend toward a greater practice of physical activity in the participants who presented a lower consumption of UPF, the differences between groups were not statistically significant. For this reason, the statistics were only adjusted by gender, as in this case there were differences that could be compromised in the objective of evaluating the oxidative and inflammatory state according to the UPF intake of these participants. Food processing can alter nutritional, structural, and chemical characteristics through the presence of artificial sweeteners, additives, and neoformed contaminants. In this way, health and satiety signaling can be altered. The biological pathways through which ultra-processed foods influence cardiovascular health may involve complex mechanisms and synergies among many compounds and characteristics of UPFs [35]. There has been evidence to suggest that the high content of additives and artificial sweeteners, such as sucralose, trans fats, and advanced glycation end-products (AGEs), in UPFs contribute to the inflammatory cascade and in the instauration of oxidative stress [36].

In terms of nutrient intake, and in accordance with a previous study, higher consumption of SFA and trans-FA was observed in the group with a greater consumption of UPFs, while the intake of fiber was significantly lower [37]. The differences in the quality of the diet between high UPF and low UPF consumers were also evident when analyzing the urinary levels of polyphenols. Thus, although the levels of polyphenols in plasma are similar between high and low UPF consumers, higher values were observed in the urine of lower UPF consumers. These results might be attributed to polyphenol fast metabolization and urine excretion, since these compounds are rapidly conjugated with the glucuronide, sulphate, and methyl groups, favouring urinary and biliary excretion, with maximal plasma levels appearing 1–2 h after ingestion [38]. According to the kinetics of the absorption, metabolism, distribution, and excretion of these compounds in the body, their half-life can vary from half an hour to 20 h, which causes the plasmatic levels to be much more stable, especially if the samples have been obtained after an overnight fast. Therefore, it is to be expected that the concentrations of these compounds are higher in the first urine of the morning than in the plasma obtained after a 12-h fast [39]. Since polyphenols predominate in healthy and fresh foods such as fruits and vegetables, the overall intake of polyphenols and their derivatives decreases when people consume significant amounts of UPFs, being poor in these bioactive substances [40]. 

Regarding oxidation status, while no differences were observed in the intake of exogenous antioxidants, there were differences in the levels of endogenous antioxidants and prooxidants. Specifically, the higher UPF consumers showed lower activity of CAT and SOD, and high xanthine oxidase (XO) levels. XO is an enzyme that converts xanthine and hypoxanthine to uric acid, generating superoxide anion (O_2_^−^), which is then catalyzed by SOD into hydrogen peroxide (H_2_O_2_) and oxygen. O_2_^−^, together with H_2_O_2_ and hydroxyl radical (OH^−^), are the main ROS that can cause cellular damage if they accumulate in high concentrations [40]. The ratio between these two enzymes could be used as a biomarker to assess the balance between ROS levels and antioxidant mechanisms. Increased XO activity may increase the creation of ROS and the degree of oxidative stress, whereas decreasing SOD activity may limit the body’s ability to neutralize ROS. Therefore, a high XO/SOD ratio could suggest a high ROS production capacity, and consequently, an imbalance between oxidative stress and antioxidant defenses. On the other hand, CAT decomposes H_2_O_2_ into water and molecular oxygen [41]. The SOD/CAT enzyme ratio could also serve to reflect the balance of the oxidation state, since a high SOD/CAT ratio could imply a greater capacity for ROS detoxification and therefore promote an antioxidant status.

Current results showed that the production of ROS by Zym-stimulated neutrophils was higher in the high UPF consumers, whereas the differences were not significant in LPS-stimulated neutrophils. Zym is an insoluble preparation of the cell wall of the fungus *Saccharomyces cerevisiae*, usually used as a fungal mimic to activate phagocytes [42]. In contrast, LPS is a complex molecule found in the cell envelope of many Gram-negative bacteria that provides a molecular pattern for the host cell receptors to elicit an immune response [43]. Zym and LPS are microbial activators that bind to toll-like receptors (TLRs), which are innate immune receptors. When neutrophils are activated with LPS, they interact with TLR4, while Zym interacts with TLR2/6, finally leading to the activation of NADPH oxidase [44]. This increase in ROS production and oxidative stress contributes to the appearance of inflammation, altering vascular function and leading to vascular disease [45]. The observed difference between high and low UPF consumers suggests that immune cells experience a higher degree of pre-activation, consequently promoting a more pro-inflammatory state [46]. In this sense, it has been shown that SFAs can activate TLR2 and TLR4, inducing their dimerization and translocation into lipid rafts in the plasma membrane, while PUFAs, particularly docosahexaenoic acid (DHA), inhibit activation. Thus, the higher intake of SFA associated with the higher intake of UPF could contribute to a state of pre-activation [47].

MPO is a protein released by leukocytes that plays a very important role as part of the innate immune system through the formation of microbicidal reactive oxidants, thus playing a crucial role in inflammation and oxidative stress [48]. Although, to date, no relationship has been established between the activity of MPO and the consumption of UPFs, the current study showed greater activity of this enzyme in the high UPF consumers. Therefore, a high UPF intake, which could displace the intake of healthy foods rich in antioxidants, could be related to this increase in activity and may be a consequence of the increase in the pro-oxidative status. 

The present findings revealed that high UPF consumers showed higher plasma levels of TNFα, IL-6, IL-15, and leptin than lower UPF consumers, suggesting a higher proinflammatory status. Accordingly, a recent study showed that high consumption of UPFs may double the risk of subclinical coronary atherosclerosis [49]. Previous studies have reported a direct association between UPF intake and a dietary pattern high in calories, sweets, refined grains, red and processed meats, snacks, and sugary beverages with serum IL-6 levels, suggesting that decreasing UPF intake can help reduce chronic inflammation [50,51]. TNFα, a protein involved in the inflammatory process, showed a positive correlation with the intake of the proinflammatory trans-FA and SFA, both related to vascular endothelial dysfunction [52]. Moreover, it has been observed that high UPF intake is connected to high leptin levels, which are linked to insulin resistance [53]. This situation is associated with a proinflammatory status, neuroinflammation, and metabolic dysfunctions, and may also affect the composition of the gut microbiota [53]. IL-15 is an inflammatory cytokine implicated in several cardiovascular diseases, such as myocardial infarction and atherosclerosis [54]. Another study carried out on adolescents showed that those who had high UPF consumption showed high levels of CRP, leptin, IL-6, and TNFα [55]. High levels of IL-15 are in accordance with another study that found an upregulation of IL-15 in atherosclerotic lesions, which may help to attract and activate T cells contributing to the progression of the disease due to its pro-inflammatory properties [56]. No significant changes were observed in MCP-1 and IL-1ß levels, and these results are in accordance with previous findings that healthy overweight or obese women with high UPF consumption showed high levels of CRP, but similar levels of MCP-1 and IL-1ß go low UPF consumers [57]. 

### Strengths and Limitations of the Study 

The main strengths of the study are that it employed a homogeneous population and, even so, the markers used made it possible to detect differences associated with UPF consumption. Despite the limitation in sample size, this study provided significant evidence of disparities in biomarker levels between high and low consumers of UPFs. These findings suggest the need for further research to fully understand the health impacts associated with UPF consumption. To improve future investigations, it is recommended to enlarge the sample size, and conduct a more detailed and specific analysis by gender, which could provide a more comprehensive understanding of how biological differences may influence the response to ultra-processed foods.

## 5. Conclusions

The current study demonstrates that despite having similar blood biochemical profiles, MetS individuals with high UPF consumption show higher pro-oxidant and pro-inflammatory profiles than low UPF consumers. These results show that although the general biochemical analyses do not show differences between UPF consumers, there is a high cardiovascular risk when inflammatory and oxidative markers are analyzed. This pro-oxidant and pro-inflammatory balance may contribute to the advancement of cardiovascular, metabolic, and inflammatory disorders, among others, even if their increase is often multifactorial. Therefore, it is necessary to develop policies aimed at decreasing the degree of food processing, as well as to encourage the use of unprocessed and local foods.

## Figures and Tables

**Table 1:** Characteristics of participants according to ultra-processed (UPF) food intake.

	Low UPF Intake (n = 46)	High UPF Intake (n = 46)	*p*-Value
Age (years)	65.1 ± 4.5	64.6 ± 4.4	0.612
Female [n (%)]	25 (54.3)	13 (28.3)	0.011
Male [n (%)]	21 (45.7)	33 (71.7)	
Anthropometrical parameters			
Weight (kg)	84.6 ± 12.0	90.6 ± 12.9	0.205
Height (cm)	161 ± 9	166 ± 8	0.068
BMI (kg/m^2^)	32.7 ± 3.3	32.8 ± 3.9	0.739
WHtR	0.687 ± 0.056	0.678 ± 0.061	0.586
Abdominal obesity (cm)	110.3 ± 9.6	112.5 ± 10.0	0.808
Systolic blood pressure (mmHg)	144 ± 19	141 ± 18	0.210
Diastolic blood pressure (mmHg)	81.6 ± 11.6	82.0 ± 10.4	0.884
Clinical parameters			
Glucose (mg/dL)	115 ± 24	121 ± 50	0.945
HbA1c (%)	6.14 ± 0.72	6.28 ± 1.50	0.951
Triglycerides (mg/dL)	153 ± 68	162 ± 83	0.802
HDL-Cholesterol (mg/dL)	45.5 ± 11.5	44.0 ± 11.8	0.963
LDL-Cholesterol (mg/dL)	116 ± 36	109 ± 30	0.605
Cholesterol total (mg/dL)	191 ± 40	184 ± 33	0.712
Uric acid (mg/dL)	6.12 ± 1.50	6.23 ± 1.37	0.611
Lifestyle parameters			
Physical activity (METs·min/week)	3734 ± 3600	2960 ± 2623	0.055
Mediterranean diet adherence (score)	8.15 ± 2.4	6.80 ± 2.03	0.015
Basal metabolism rate (kcal)	1628 ± 278	1772 ± 280	0.310
Ingested calories (Kcal/day)	2385 ± 809	2575 ± 658	0.857
DII	−0.029 ± 1.73	0.721 ± 1.89	0.038
Drug intake			
Antidiabetic [n (%)]	17 (37%)	14 (30%)	0.508
Antihypertensive [n (%)]	38 (83%)	37 (80%)	0.788
Lipid-lowering [n (%)]	24 (52%)	30 (65%)	0.204

Results are expressed as mean ± standard deviation (SD). *p*-values by Student’s *t*-test adjusted by sex. Pearson χ² test was performed in dichotomous variables. Abbreviations: BMI: body mass index; DII: dietary inflammatory index; HbA1c: glycated haemoglobin A1c; MET: metabolic equivalent of task; WHtR: waist-to-height ratio.

**Table 2:** Nutrient intake according to UPF intake.

	Low UPF Intake (n = 46)	High UPF Intake (n = 46)	*p*-Value
Macronutrients			
Carbohydrates (g/day)	246.6 ± 98.1	265.3 ± 74.9	0.768
Proteins (g/day)	94.0 ± 28.0	97.0 ± 24.4	0.722
Lipids (g/day)	101.3 ± 37.0	113.8 ± 37.7	0.475
Micronutrients			
MUFAs (g/day)	51.2 ± 20.3	56.7 ± 20.1	0.609
PUFAs (g/day)	17.2 ± 7.5	19.1 ± 16.8	0.567
SFA (g/day)	25.9 ± 9.9	30.9 ± 11.3	0.014
Trans FA (g/day)	0.570 ± 0.307	0.759 ± 0.472	0.013
w-6 FA (g/day)	13.4 ± 6.2	15.0 ± 7.1	0.522
w-3 FA (g/day)	0.795 ± 0.464	0.755 ± 0.369	0.524
Cholesterol (mg/day)	390.0 ± 178.6	424.5 ± 120.3	0.620
Folic acid (µg/day)	357.7 ± 119.7	328.9 ± 107.9	0.154
Fiber (g/day)	28.5 ± 10.3	25.2 ± 8.9	0.048
Vitamins			
Vitamin A (µg/day)	1163 ± 613	1331 ± 757	0.339
Vitamin C (mg/day)	217 ± 88	213 ± 95	0.783
Vitamin D (µg/day)	5.57 ± 3.74	5.13 ± 2.43	0.492
Vitamin E (mg/day)	10.3 ± 3.9	11.2 ± 3.5	0.529
Minerals			
Phosphor (mg/day)	1645 ± 463	1730 ± 425	0.890
Magnesium (mg/day)	424 ± 130	421 ± 122	0.437
Iron (mg/day)	16.9 ± 5.6	17.2 ± 4.7	0.672
Iodine (mg/day)	234.1 ± 119.5	228.1 ± 148.7	0.432
Potassium (mg/day)	4555 ± 1299	4416 ± 1251	0.324
Calcium (mg/day)	941.8 ± 287.6	988.7 ± 344.7	0.958
Sodium (mg/day)	2524 ± 1188	2662 ± 934	0.751
Selenium (mg/day)	115.0 ± 40.1	112 ± 32.8	0.306
Zinc (mg/day)	12.2 ± 3.9	12.6 ± 3.3	0.778
Glycaemic load	140.1 ± 64.0	151.4 ± 49.1	0.806
Glycaemic index	55.8 ± 5.4	56.5 ± 4.6	0.772
Alcohol (g/day)	15.8 ± 20.9	15.3 ± 18.0	0.167

Results are expressed as mean ± standard deviation (SD). *p*-values by Student’s *t*-test adjusted by sex. Abbreviations: MUFA: monounsaturated fatty acid; PUFA: polyunsaturated fatty acid; SFA: saturated fatty acid; trans FA: trans- fatty acid; ω-3 FA: omega-3 fatty acid.

**Table 3:** Oxidative stress and inflammatory parameters according to UPF intake.

	Low UPF Intake (n = 46)	High UPF Intake (n = 46)	*p*-Value
Plasma markers			
CAT activity (k/L)	56.4 ± 29.0	46.5 ± 17.4	0.047
SOD activity (pkat/L)	180.1 ± 88.4	136.6 ± 71.4	0.018
MPO activity (µkat/mL)	53.3 ± 28.9	67.6 ± 31.2	0.016
XO levels (µg/L)	0.395 ± 0.211	0.535 ± 0.449	0.030
MDA levels (nM)	1.06 ± 0.63	1.15 ± 0.69	0.253
TNFα levels (pg/mL)	3.17 ± 1.83	4.37 ± 1.71	0.003
IL-1ß levels (pg/mL)	19.1 ± 42.2	22.5 ± 48.9	0.366
IL-6 levels (pg/mL)	4.23 ± 3.11	5.78 ± 3.75	0.022
IL-15 levels (pg/mL)	7.39 ± 3.24	10.2 ± 5.82	0.048
INF-γ levels (pg/mL)	5.92 ± 1.75	6.22 ± 1.85	0.330
MCP-1 levels (pg/mL)	234.5 ± 89.0	232.8 ± 78.9	0.462
Resistin levels (ng/mL)	5.60 ± 6.43	6.40 ± 8.39	0.385
Ghrelin levels (pg/mL)	302.1 ± 53.9	313.0 ± 57.2	0.298
Leptin levels (ng/mL)	10.3 ± 7.6	15.1 ± 16.3	0.044
Polyphenol levels (nM)	0.058 ± 0.020	0.057 ± 0.016	0.389
Urine markers corrected by creatinine			
MDA levels (mM/mM)	85.0 ± 53.1	108.9 ± 94.4	0.133
Polyphenol levels (mM/mM)	13.0 ± 3.97	10.9 ± 3.17	0.027
OxoGuo levels (nM/mM)	1.84 ± 0.39	1.91 ± 0.47	0.279
OxodG levels (nM/mM)	1.31 ± 0.43	1.43 ± 0.55	0.189

Results are expressed as mean ± standard deviation (SD). *p*-values by Student’s *t*-test adjusted by sex. Abbreviations: CAT: catalase; SOD: superoxide dismutase; MPO: myeloperoxidase; XO: xanthine oxidase; MDA: malondialdehyde; TNFα: tumour necrosis factor α; IL-1β: interleukine-1β; IL-6: interleukine-6; IL-15: interleukine-15; INF-γ: interferon-γ; MCP-1: monocyte chemoattractant protein 1; 8-oxodG: 8-oxo-7,8-dihydro-guanosine; 8-oxoGuo: 8-oxo-7,8-dihydroguanosine.

## Data Availability

There are restrictions on the availability of data for this trial, due to the signed consent agreements around data sharing, which only allow access to external researchers for studies following the project purposes. Researchers wishing to access the trial data used in this study can make a request to the corresponding author: pep.tur@uib.es.
